# Lessons from the COVID-19 Pandemic

**DOI:** 10.3390/jcm12185791

**Published:** 2023-09-06

**Authors:** Marco Giani, Matteo Pozzi, Roberto Rona

**Affiliations:** 1 Department of Medicine and Surgery, University of Milano-Bicocca, 20900 Monza, Italy; matteo.pozzi@irccs-sangerardo.it; 2 Department of Emergency and Intensive Care, IRCCS San Gerardo dei Tintori, 20900 Monza, Italy; roberto.rona@irccs-sangerardo.it

The COVID-19 pandemic was an unprecedented global crisis that significantly impacted around the world. The dramatic surge of COVID-19 patients forced healthcare systems to restructure intensive care departments, including teams, spaces, and clinical organization. The pandemic has brought significant changes in the way intensive care units (ICUs) operate and has highlighted several crucial aspects that require attention and improvement.

The current Special Issue of the *Journal of Clinical Medicine* is dedicated to the “transition” at the end of the pandemic, allowing us to step away from COVID-19 into a future with a more modern, personalized, and “human” ICU care. The issue gathered eight papers on this topic, which provide useful insights into the potential transformations that can enhance our preparedness for future challenges.

From a clinical point of view, ICU physicians had to face new, original challenges. Healthcare workers had to treat a disproportionate number of critically ill COVID-19 patients, which they were not accustomed to managing, both in terms of treatment and scale. Due to the exposure risk, many routine procedures were burdened by an extraordinary rise in technical difficulties and safety issues. In their paper [1], Conoscenti et al. explored the impact of an organizational model adopted during the pandemic on the perceived safety of the ICU staff. In the context of an Italian hospital with a COVID-19 dedicated intensive care unit, a bundle of organizational, technical, and structural interventions aimed at the protection of the staff was perceived as appropriate by more than 90% of the respondents.

The theme of transition to more personalized care was addressed in several papers included in the Special Issue.

Di Pierro et al. described the application of Electrical Impedance Tomography (EIT) to set positive end-expiratory pressure (PEEP) in patients with ARDS on veno-venous extracorporeal support [2]. EIT was feasible and allowed the “tailoring” of the PEEP level in these patients. Severe COVID-19-related ARDS showed respiratory characteristics similar to severe “classical” ARDS, thus allowing them to generalize these findings to patients with ARDS from other etiologies.

Specific innovative treatments for respiratory failure were evaluated. Ruaro and colleagues reported [3] the use of large volumes of surfactant via bronchoalveolar lavage to maximize its distribution in the respiratory tract. This treatment was used in two patients and, despite its unproven benefit to date, may be a promising strategy for the management of ARDS.

To better understand the patient disease trajectory, Salton et al. studied the prognostic value of cytokine patterns [4]. The trajectory of cytokine levels in patients treated with noninvasive ventilation (NIV) was compared to patients who required invasive mechanical ventilation. Patients in the latter group showed higher inflammation levels than the NIV group, which may indicate either a more severe disease or a higher resistance to glucocorticoids. The same research group summed up [5] the state-of-the-art on the topic of monitoring patients treated with noninvasive ventilation. Great attention was paid to distinguishing the different ARDS phenotyping, highlighting the need for personalization of care in these patients.

New prognostic models for critical patients were also evaluated. Moisa et al. [6] tested a modified version of the SOFA score adapted to COVID-19 patients. The COVID-SOFA score performed better than the SOFA score alone for 28-day mortality prediction.

The COVID-19 pandemic may have affected the outcomes and the recovery trajectory of non-COVID-19 patients.

Juárez-Vela et al., in their cross-sectional study [7], compared two cohorts of patients (before and during the pandemic wave) admitted to Spanish ICUs with a diagnosis of polytrauma to evaluate a potential impact on this population of healthcare system reorganization due to COVID-19. Transfusion practices were significantly changed during the study period, with an increase in the use of blood products, whereas mortality did not change.

In their multicenter study [8] on a prospective cohort of 237 patients, Kang and colleagues analyzed the prevalence of post-intensive care syndrome (PICS) in non-COVID-19 ICU survivors during the pandemic in South Korea. Scores differed significantly according to whether participants completed follow-up before or after December 2020, when COVID-19 rapidly spread in their country. In patients evaluated during the pandemic, anxiety, depression, post-traumatic stress disorder, and cognition scores were significantly worse at 12 months post-discharge.

In conclusion, the COVID-19 pandemic provided valuable lessons for ICUs worldwide: Implementation of personalized medicine for critically ill patients [2,5], focus on long-term outcomes [8], revision of organizational models [1], research for new treatments [3], and improved outcome prognostication [5,6]. By applying these lessons, ICUs can transform their practices, ultimately improving patient care and better protecting the well-being of healthcare professionals. It is now crucial to seize this opportunity to strengthen healthcare systems and ensure that ICUs are well-equipped to face future challenges.
